# Supplementary material for: Identification of amino acid residues in polymerase PB2 responsible for differential replication and pathogenicity of avian influenza virus H5N1 isolated from human and cattle in Texas, US
Source: Emerg Microbes Infect. 2025 Aug 1;14(1):2542247. doi: 10.1080/22221751.2025.2542247 (PMC12372494; doi:10.1080/22221751.2025.2542247)
Supplement: Supplementary Table.docx [file TEMI_A_2542247_SM5392.docx]

1. **Supplementary** **Tables**

**Supplementary** **Table 1. Primers used to generate rHPbTX and rHPhTX PB2 mutants**

| Variation (aa)  hTX → bTX | Primer name | Primer sequence (5`---3`) | Reference |
| --- | --- | --- | --- |
| 362G | H5N1_TPB2_A1112G_F | GTACATGAGGGGTATGAAGGGTTCACTATGGTTGGAAG | ^22^ |
|  | H5N1_TPB2_ A1112G_R | CTTCCAACCATAGTGAACCCTTCATACCCCTCATGTAC |  |
| 627E | H5N1_TPB2_A1906G_F | CTTTGCTGCTGCCCCACCGGAACAAAGTAGACTGCAATTC |  |
|  | H5N1_TPB2_ A1906G_R | GAATTGCAGTCTACTTTGTTCCGGTGGGGCAGCAGCAAAG |  |
| 631L | H5N1_TPB2_A1918C_F | CCCACCGAAACAAAGTAGACTGCAATTCTCCTCTCTGAC |  |
|  | H5N1_TPB2_ A1918C_R | GTCAGAGAGGAGAATTGCAGTCTACTTTGTTTCGGTGGG |  |
| Variation (aa)  bTX → hTX | Primer name | Primer sequence (5`---3`) | Reference |
| 362E | H5N1_MBPB2_G1112A_F | CATGAGGGGTATGAAGAGTTCACTATGGTTGG | This study |
|  | H5N1_MBPB2_ G1112A _R | CCAACCATAGTGAACTCTTCATACCCCTCATG |  |
| 627K | H5N1_MBPB2_G1906A_F | GCTGCTGCCCCACCGAAACAAAGTAG |  |
|  | H5N1_MBPB2_ G1906A_R | CTACTTTGTTTCGGTGGGGCAGCAGC |  |
| 631M | H5N1_MBPB2_C1918A_F | AACAAAGTAGAATGCAATTCTCCTCTC |  |
|  | H5N1_MBPB2_ C1918A_R | GAGAGGAGAATTGCATTCTACTTTGTT |  |
